# Construction of a MicroRNA-Based Nomogram for Prediction of Lung Metastasis in Breast Cancer Patients

**DOI:** 10.3389/fgene.2020.580138

**Published:** 2021-02-19

**Authors:** Leyi Zhang, Jun Pan, Zhen Wang, Chenghui Yang, Jian Huang

**Affiliations:** ^1^Key Laboratory of Tumor Microenvironment and Immune Therapy of Zhejiang Province, Second Affiliated Hospital, Zhejiang University School of Medicine, Hangzhou, China; ^2^Cancer Institute (Key Laboratory of Cancer Prevention Intervention, National Ministry of Education), Second Affiliated Hospital, Zhejiang University School of Medicine, Hangzhou, China; ^3^Department of Breast Surgery, Second Affiliated Hospital, Zhejiang University School of Medicine, Hangzhou, China

**Keywords:** breast cancer, lung metastasis, microRNA, nomogram, the cancer genome atlas, METABRIC dataset, risk score

## Abstract

The lung is one of the most common sites of distant metastasis in breast cancer (BC). Identifying ideal biomarkers to construct a more accurate prediction model than conventional clinical parameters is crucial. MicroRNAs (miRNAs) data and clinicopathological data were acquired from the Molecular Taxonomy of Breast Cancer International Consortium (METABRIC) database. miR-663, miR-210, miR-17, miR-301a, miR-135b, miR-451, miR-30a, and miR-199a-5p were screened to be highly relevant to lung metastasis (LM) of BC patients. The miRNA-based risk score was developed based on the logistic coefficient of the individual miRNA. Univariate and multivariate logistic regression selected tumor node metastasis (TNM) stage, age at diagnosis, and miRNA-risk score as independent predictive parameters, which were used to construct a nomogram. The Cancer Genome Atlas (TCGA) database was used to validate the signature and nomogram. The predictive performance of the nomogram was compared to that of the TNM stage. The area under the receiver operating characteristics curve (AUC) of the nomogram was higher than that of the TNM stage in all three cohorts (training cohort: 0.774 vs. 0.727; internal validation cohort: 0.763 vs. 0.583; external validation cohort: 0.925 vs. 0.840). The calibration plot of the nomogram showed good agreement between predicted and observed outcomes. The net reclassification improvement (NRI), integrated discrimination improvement (IDI), and decision-curve analysis (DCA) of the nomogram showed that its performances were better than that of the TNM classification system. Functional enrichment analyses suggested several terms with a specific focus on LM. Subgroup analysis showed that miR-30a, miR-135b, and miR-17 have unique roles in lung metastasis of BC. Pan-cancer analysis indicated the significant importance of eight predictive miRNAs in lung metastasis. This study is the first to establish and validate a comprehensive lung metastasis predictive nomogram based on the METABRIC and TCGA databases, which provides a reliable assessment tool for clinicians and aids in appropriate treatment selection.

## Introduction

Breast cancer (BC) is the most common cancer diagnosed (excluding skin cancers) and is the second leading cause of cancer death among United States women (DeSantis et al., 2019) and worldwide. Most BC-related deaths are caused by distant metastases, which become lethal even after the primary lesion being removed (Knott et al., 2018). BC tends to metastasize distantly to the bone, brain, liver, lung, and distant lymph nodes. Lung metastases particularly tend to occur within the initial 5 years of BC diagnosis and significantly impact patients’ prognosis (Medeiros and Allan, 2019). Therefore, it is of great clinical importance to select patients who are prone to have lung metastasis so that they can benefit from prevention treatment and early diagnosis.

Currently, the traditional tumor node metastasis (TNM) staging system is a standard tool for risk evaluation in clinical practice. However, BC patients with the same stage can have varying clinical outcomes (Wang et al., 2019). The TNM staging system is mainly based on anatomical information, which fails to incorporate important pathological parameters and biological changes that happened in BC. The mechanisms of the lymphatic dissemination and hematogenous dissemination are different, which may be one of the reasons for the poor metastasis prediction ability of the TNM staging system. Hence, new methods to identify patients who are likely to have lung metastasis are needed.

MicroRNAs (miRNAs) are small, non-coding single-stranded RNAs (18–25 nucleotides) and negatively regulate gene expression by binding to complementary sequences in the 3' untranslated region (3' UTR) of mRNAs (Lin and Gregory, 2015). Accumulating evidence suggests that miRNAs play critical roles in various physiological and pathological processes, including many proposed mechanisms of cancer metastasis (Pencheva and Tavazoie, 2013). Previous studies have presented the association of certain miRNAs with lung metastasis, including miR-629-3p, miR-106b-5p, and so on (Schrijver et al., 2017; Wang et al., 2017). However, due to the biological heterogeneity of BC, a comprehensive prediction model incorporating multiple biomarkers, rather than a single parameter, can improve predictive accuracy. Nomograms constructed on the basis of known predictive variables are being widely used to predict the specific outcome for an individual patient (Iasonos et al., 2008). There have been reports that clinical variables-based nomogram and miRNA signature could be used to predict distant metastasis in BC patients (Delpech et al., 2015; Rohan et al., 2019), yet there is no literature concerning comprehensive lung metastasis prediction model. We hypothesized that our new model based on the combination of predictive miRNAs and clinicopathological variables could improve the accuracy in predicting lung metastasis and prolong survival in BC patients.

Therefore, the purpose of this study was to establish and validate a comprehensive nomogram that incorporated both the miRNAs signature and clinical-related risk features for the individual prediction of lung metastasis status of BC patients. The new prediction model was compared with the traditional TNM staging system in order to determine its reliability. Aiding with this model, clinicians might be able to evaluate the lung metastasis risk of BC patients, thus choosing appropriate medical examinations and optimizing therapeutic regimen.

## Materials and Methods

### Datasets Selection and Data Processing

To identify lung metastasis-related miRNA and mRNA in BC, public datasets with matched miRNA, mRNA, and clinical data were used in this study. A European Genome-phenome Archive (RRID: SCR_004944),^1^ EGAS00000000122 (Molecular Taxonomy of Breast Cancer International Consortium, METABRIC miRNA landscape; Curtis et al., 2012; Dvinge et al., 2013), contains a total of 1,302 BC patients with matched mRNA (EGAD00010000434) and miRNA (EGAD00010000438) data. The inclusion criteria included: (1) samples had lung metastasis or no metastasis (NM); (2) samples had both mRNA and miRNA expression data; and (3) samples had intact clinical data. Around 439 patients were selected in subsequent analysis. Among them (*n* = 439), 327 samples were randomly assigned as a training cohort and the rest were assigned as an internal validation cohort based on a computer number generator (Supplementary Table S1). About 449 of 1,109 BC patients from The Cancer Genome Atlas (TCGA) dataset (RRID: SCR_003193) were selected according to the same inclusion criteria as an external validation cohort (Network, 2012; Supplementary Table S1).^2^ The method of acquisition and application complied with the guidelines and policies. It is not necessary to obtain informed patient consent for data obtained from the METABRIC and TCGA databases since they do not include information that can be used to identify individual patients.

### Development of a miRNA-Based Risk Score

Among the 439 BC patients in the METABRIC dataset, two subsets of patients were defined based on their metastasis status: a lung metastasis group (those who had lung metastasis) and an NM group (those who did not report metastasis until the last follow-up). We identified 853 miRNAs annotated in the METABRIC dataset, and differentially expressed miRNAs (DEmiRNAs) between the two groups were identified using the *LIMMA* package of R (Ritchie et al., 2015; LIMMA, RRID: SCR_010943). Of the top 20 DEmiRNAs with the most significant foldchanges, four miRNAs were dropped from highly correlated pairs (*r* > 0.8, Wei and Simko, 2017). The least absolute shrinkage and selection operator (LASSO) method (Friedman et al., 2010) was used to select the most useful predictive miRNAs from the 16 lung metastasis-related DEmiRNAs in the training cohort and constructed an eight-miRNA based risk score for predicting lung metastasis status of BC patients in the training set. The risk score was calculated for each patient *via* a linear combination of selected miRNAs that were weighted by their respective coefficients.

$$Riskscore=\sum_{i=1}^{8}\beta_{i}\times Exp_{i}$$

An optimal cut-off point was determined using receiver operating characteristic (ROC) curve, to classify samples into low (≤0.168) and high risk (>0.168) group. The Kaplan-Meier (KM) survival analysis with a log-rank test was implemented to compare the survival difference between the two groups (Kassambara et al., 2017). Then KM analysis with the log-rank test was also implemented to show the relationship between the expression of predictive miRNAs and prognosis in external validation cohort.

### Construction and Validation of miRNA-Based LM Predictive Nomogram

Univariate logistic regression analysis was performed to compare the predictive power of the eight-miRNA risk score and clinical parameters including age at diagnosis, tumor size, TNM stage, grade, estrogen receptor (ER) status, progesterone receptor (PR) status, human epidermal growth factor receptor 2 (HER2) status, and hormone therapy. Furthermore, we used a multivariate logistic regression analysis to determine whether the eight-miRNA risk score could be an independent predictive factor for lung metastasis in BC patients. Other clinical parameters with values of *p* less than 0.1 in the univariate logistic regression analysis were also incorporated in the analysis. A composite nomogram was constructed based on all independent predictive parameters screened by multivariate logistic regression analysis above to predict the rate of lung metastasis (Harrell, 2013), and to be a graphic representation of the prediction model.

The ROC curves were plotted to assess the sensitivity and the specificity of independent predictive parameters including eight-miRNA signature, age at diagnosis, TNM stage, and miRNA-based nomogram in predicting lung metastasis (Sing et al., 2005). The area under the receiver operating characteristics curve (AUC) was also calculated to make a comparison for the discriminatory ability of the above predictive parameters. Calibration curves were implemented to assess the calibration ability of the miRNA-based nomogram, accompanied by the Hosmer-Lemeshow test (Kramer and Zimmerman, 2007). The predicted and observed outcomes of the nomogram could be compared in the calibration curve, while the 45-degree diagonal line represented the ideal prediction. The net reclassification improvement (NRI) and integrated discrimination improvement (IDI) were used to quantify the improvement in sensitivity and specificity offered by our miRNA-based nomogram compared to the TNM staging system (Kundu et al., 2011). NRI was based on reclassification tables composed of patients with and without events and could quantify the correct reclassification in categories (Pencina et al., 2011). IDI summarized the extent to which a new model increased risk in patients with events and decreased risk in patients without events (Pencina et al., 2008; Chipman and Braun, 2017). We used decision-curve analysis (DCA) to test the clinical applicability of our miRNA-based nomogram model by quantifying the net benefits at different threshold probabilities. DCA was conducted by adding the benefits (true positives) and subtracting the harms (false positives; Vickers and Elkin, 2006; Vickers et al., 2008).

### Identification of Potential Targets for Predictive miRNAs and Construction a miRNA-mRNA Network Associated With Lung Metastasis

The target genes of eight predictive miRNAs were first predicted and analyzed using miRWalk3.0 (RRID: SCR_016509; Sticht et al., 2018),^3^ miRDB (RRID: SCR_010848; Chen and Wang, 2020),^4^ TargetScan (RRID: SCR_010845; Nam et al., 2014),^5^ and miRTarBase (RRID: SCR_017355; Chou et al., 2018).^6^ An mRNA would be considered as a target of a miRNA if the mRNA was predicted to be the target in all three *in silico* prediction algorithms (miRWalk, miRDB, and miRTarBase) or could be found in a experimentally validated database (miRTarBase). We also acquired matched mRNA transcriptome data (RRID: SCR_004944, EGAD00010000434) of the patients enrolled in the analysis of identifying DEmiRNAs.^7^ 3,791 differentially expressed mRNAs (DEmRNAs) between the lung metastasis group and no metastasis group were identified using the *LIMMA* package of R. CytoHubba plugin (RRID: SCR_017677) in Cytoscape (RRID: SCR_003032) was used to predict the hub genes among the target genes of upregulated or downregulated miRNAs (Chin et al., 2014).^8^ miRNA-mRNA networks were also visualized with the Cytoscape software.

### Functional Enrichment Analysis of Target Genes of Predictive miRNAs

For the screened overlapped target genes of each miRNA separately or hub genes for upregulated or downregulated miRNAs, gene ontology (GO) enrichment analysis and Kyoto Encyclopedia of Genes and Genomes (KEGG) pathways analysis were performed (clusterProfiler, RRID: SCR_016884; Yu et al., 2012; Walter et al., 2015). Statistically significant GO and KEGG terms (*p* < 0.05) related to cancer and metastasis were identified.

### Identification of miRNAs Unique to Lung Metastasis or BC

MicroRNA transcriptome data of BC patients from the TCGA dataset were selected to perform two differential miRNA expression analyses between different subgroups of BC patients. Around 48 DEmiRNAs between patients with lung metastasis only and patients with distant metastasis (except for the lung) were identified using the DESeq2 package of R (DESeq2, RRID: SCR_015687; Love et al., 2014). Around 90 DEmiRNAs between patients with distant metastasis (except for the lung) and patients without metastasis were identified.

The miRNA expression data and corresponding clinical data of the patients of six cancer types [adrenocortical carcinoma (ACC), bladder urothelial carcinoma (BLCA), sarcoma (SARC), skin cutaneous melanoma (SKCM), cervical squamous cell carcinoma and endocervical adenocarcinoma (CESC), and stomach adenocarcinoma (STAD)] were downloaded from the TCGA database. DEmiRNAs between patients with lung metastasis and patients without metastasis were identified in each type of cancer using the DESeq2 package of R.

### Statistical Analysis

All the statistical analyses were performed with the SPSS software (RRID: SCR_002865) and R software (version 4.0.0; RRID: SCR_001905).^9^^,^^10^ A two-sided probability value of *p* < 0.05 was considered to be statistically significant.

## Results

### Demographic and Clinicopathological Characteristics

A total of 479 BC patients from METABRIC and 449 BC patients from TCGA were included in this study. Baseline clinical and pathological characteristics of the study participants in the training and two validation cohorts were listed in Table 1. The median age of patients was 61.11, 60.57, and 60 years in the training and two validation cohorts, respectively. The rates of lung metastasis were 8.26, 7.24, and 3.56% in the training and two validation cohorts, respectively.

**Table 1:** Demographics of the samples chosen for the study.

Variables	Training cohort (*n* = 327)	Internal validate cohort (*n* = 152)	External validate cohort (*n* = 449)
Median age at diagnosis in years (IQR)	61.11 (51.09–68.99)	60.57 (50.94–70.25)	60.00 (71.00–67.00)
Median follow up time from diagnosis in days (IQR)	3,318 (1916–4,719)	3,144 (1781–4,479)	343.5 (114–1,108)
Lung metastasis status			
No metastasis	300	141	433
Lung metastasis	27	11	16
Pam50 subtype			
Luminal A	151	78	205
Luminal B	83	37	66
HER2	26	7	21
Basal like	46	22	100
Normal breast-like	21	8	14
Unknown	0	0	43
TNM stage			
1	203	93	152
2	114	58	281
3	8	1	14
4	2	0	2
ER status			
Positive	259	118	304
Negative	68	34	124
Unknown	0	0	21
PR status			
Positive	187	87	270
Negative	142	65	156
Unknown	0	0	23
HER2 status			
Positive	41	9	53
Negative	286	143	248
Unknown			148
Menopausal state			
Pre	71	32	84
Post	256	120	312
Peri	0	0	19
Unknown	0	0	34
Vital status			
Alive	198	86	435
Dead	129	66	14

PAM50, prediction analysis of microarray 50; ER, estrogen receptor; PR, progesterone receptor; HER2, human epithelial growth factor receptor 2; and TNM, the tumor node metastasis.

### Identification Candidate Lung Metastasis-Related miRNAs in the Training Cohort

**Figure 1:**
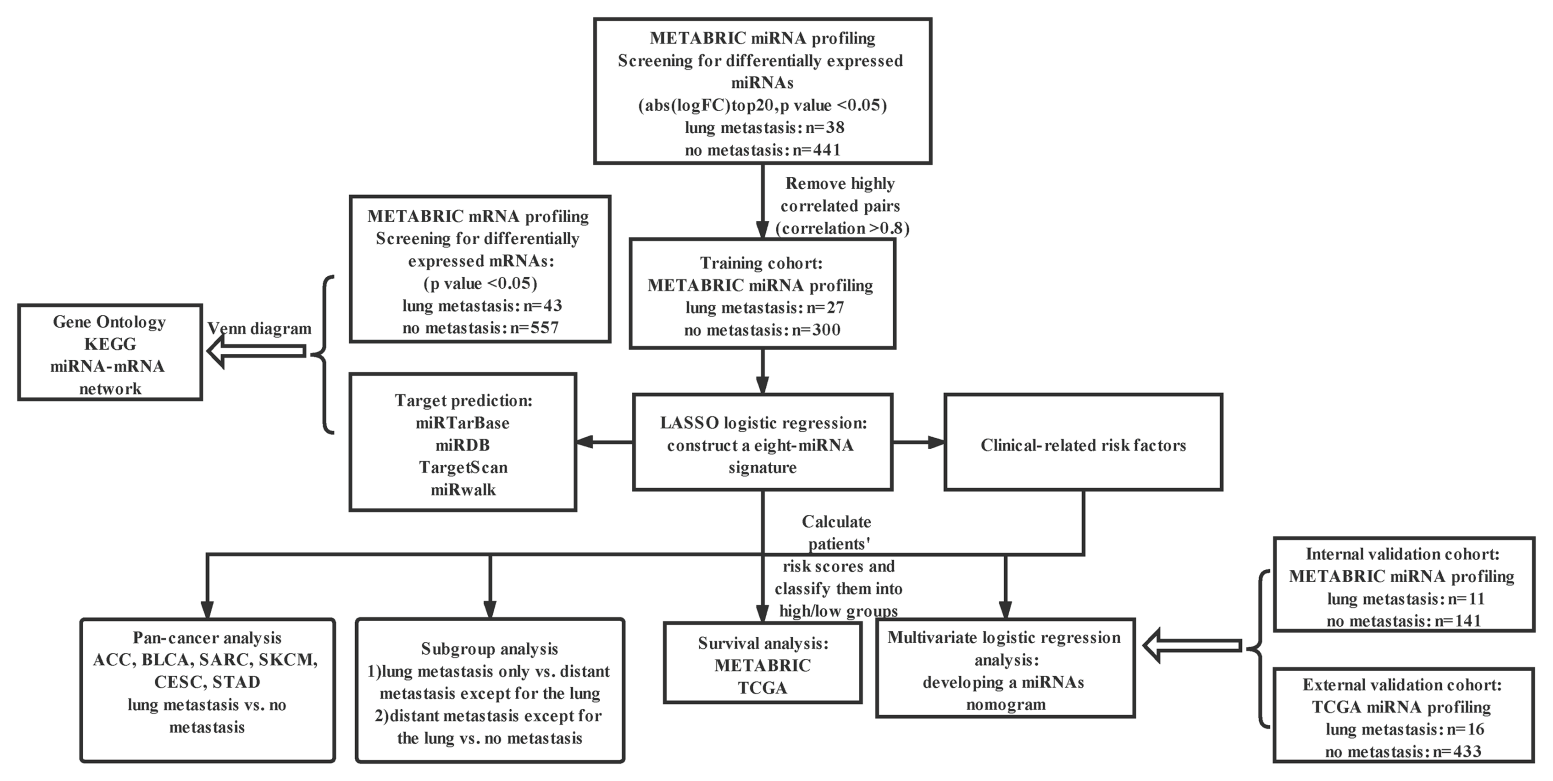
Study design. METABRIC, molecular taxonomy of BC international consortium; LASSO, the least absolute shrinkage and selector operation; KEGG, Kyoto encyclopedia of genes and genomes; Abs, absolute value; FC, fold change; miRNA, microRNA; and TCGA, the cancer genome atlas.

**Figure 2:**
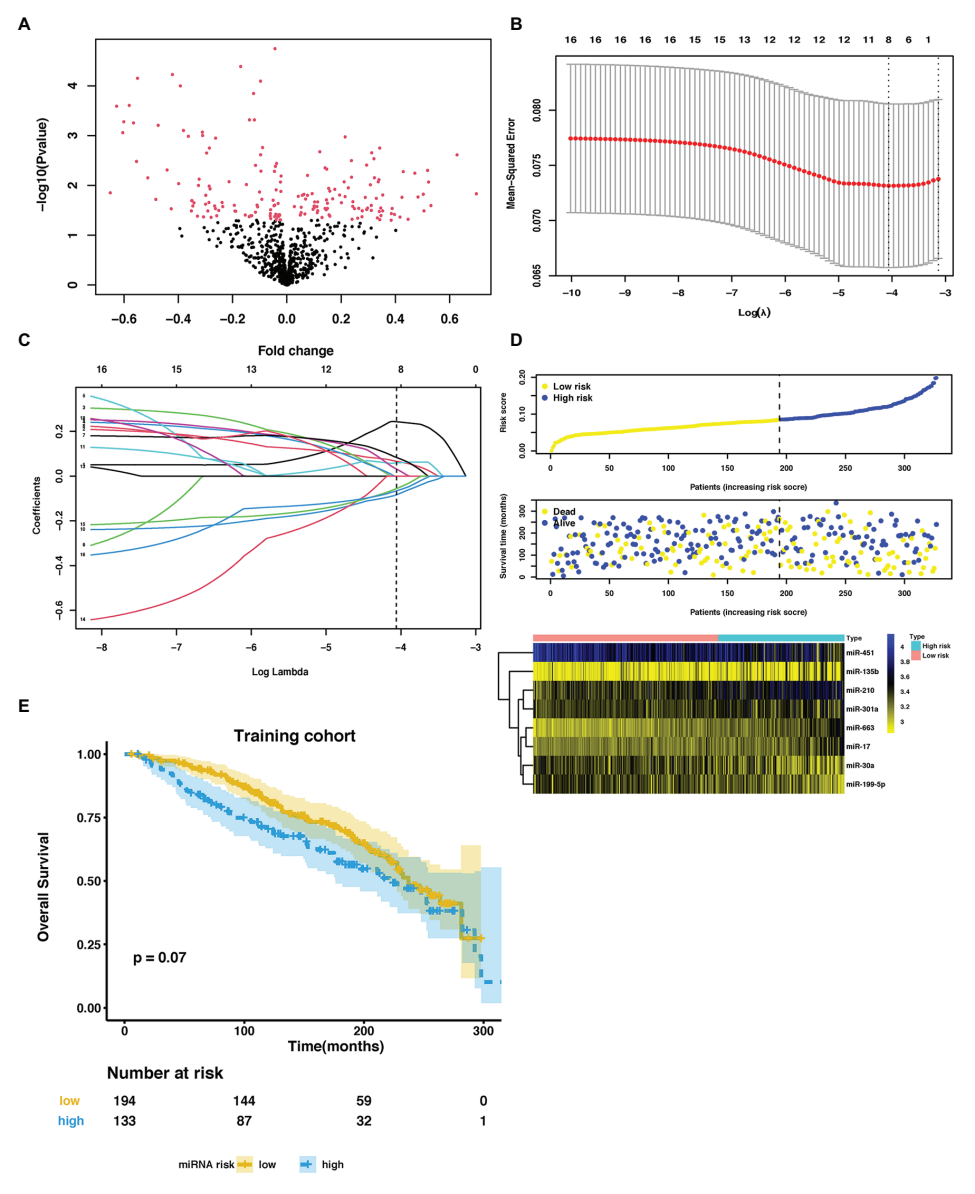
Parameter selection to develop an eight-miRNA signature to distinguish lung metastasis status of breast cancer. **(A)** Volcano plot of miRNAs expression in the METABRIC dataset. **(B)** 3-fold cross-validation for parameter selection *via* minimum criteria in the LASSO model. Two dotted vertical lines were drawn at the optimal values by using the minimum criteria (the value of lambda that gives a minimum mean cross-validated error) and the one SE of the minimum criteria (the value of lambda that gives one SE away from the minimum error). **(C)** LASSO coefficient profiles of the 16 LM-related differentially expressed miRNAs (DEmiRNAs) in the training cohort. Each curve corresponds to a miRNA. The coefficient profile plot was against the log (lambda) sequence. The dotted vertical line was drawn at the value lambda = 0.01718646 selected by using 3-fold cross-validation *via* minimum criteria, where optimal lambda resulted in eight nonzero coefficients. **(D)** The distribution of risk score, overall survival (OS), vital status, and the expression profiles of eight-miRNA in the training cohort. **(E)** Kaplan-Meier (KM) curves of OS of breast cancer patients stratified by eight-miRNA risk score in the training cohort. METABRIC, molecular taxonomy of breast cancer international consortium; LASSO, the least absolute shrinkage and selector operation; miRNA, microRNA; LM, lung metastasis; and DEmiRNAs, differentially expressed miRNAs.

The METABRIC dataset includes 1,302 BC samples, of which 479 (36.79%) of them reached the inclusion criteria for the analysis of identifying DEmiRNAs. About 327 samples were randomly assigned as a training cohort and the rest were assigned as the internal validation cohort based on a computer number generator. The flow chart of the study design was showed in Figure 1. A total of 184 miRNAs (*p* < 0.05) were identified to be differentially expressed between patients with lung metastasis and patients without metastasis (Figure 2A; Supplementary Table S2). Around 20 most significantly upregulated and downregulated miRNAs were selected to conduct correlation analysis (upregulated in lung metastasis patients: miR-663, miR-210, miR-1,202, miR-1973, miR-17, miR-18a, miR-301a, miR-135b, miR-20a, miR-17*; down-regulated in lung metastasis patients: miR-451, miR-26b, miR-199b-5p, miR-30a*, miR-10a, miR-10b, miR-30a, miR-199a-3p, miR-199a-5p, and miR-99a; Supplementary Figure S1). Four miRNAs (miR-30a*, has-miR-199a-3p, miR-99a, and miR-18a) were dropped from highly correlated pairs (*r* > 0.8) to reduce multicollinearity and improve stability for subsequent model selection.

### Development of an Eight-miRNA Signature to Distinguish Lung Metastasis Status in BC Patients

In the training cohort, we used LASSO-based logistic regression and identified eight miRNAs from the 16 DEmiRNAs, which were as follows: miR-663, miR-210, miR-17, miR-301a, miR-135b, miR-451, miR-30a, and miR-199a-5p (Figures 2B,C). The eight-miRNA based risk score was calculated based on their logistic coefficients. An optimal cut-off point was determined according to ROC. We then divided samples into a low-risk (risk score ≤ 0.168) and a high-risk (risk score > 0.168) group. The distributions of the miRNA-based risk score, overall survival (OS), OS status, and the expression profiles of eight miRNAs in the training cohort were shown in Figure 2D. The five risky upregulated miRNAs identified in lung metastasis cases exhibited high expression in the high-risk group and the three protective downregulated miRNAs had high expression in the low-risk group. And the patients with higher risk scores tended to have poorer prognoses, yet failed to reach a significant level (*p* = 0.078) (Figure 2E). Age stratified analysis indicated that miRNAs-based risk score predicted prognosis well in people aged 45–70 years (Supplementary Figure S2).

### Establishment of a Nomogram for Predicting Lung Metastasis Status Incorporating miRNAs Signature and Clinical-Related Factors

**Figure 3:**
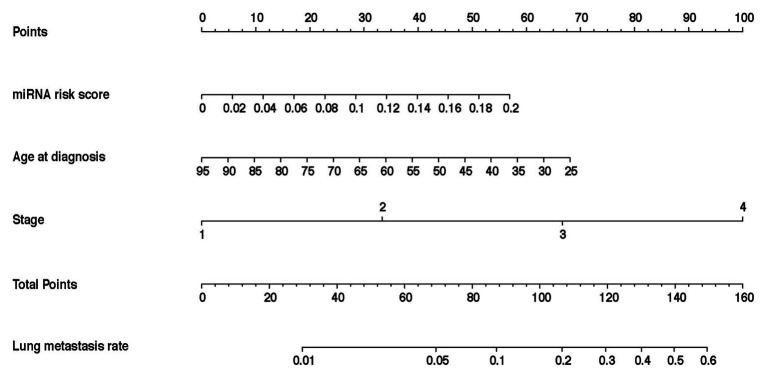
Development and assessment of the miRNA-based nomogram. Constructed a miRNA-based nomogram to predict LM for BC patients in the training cohort, with age at diagnosis, stage, and eight-miRNA signature incorporated. LM, lung metastasis; BC, breast cancer; and miRNA, micro RNA.

**Figure 4:**
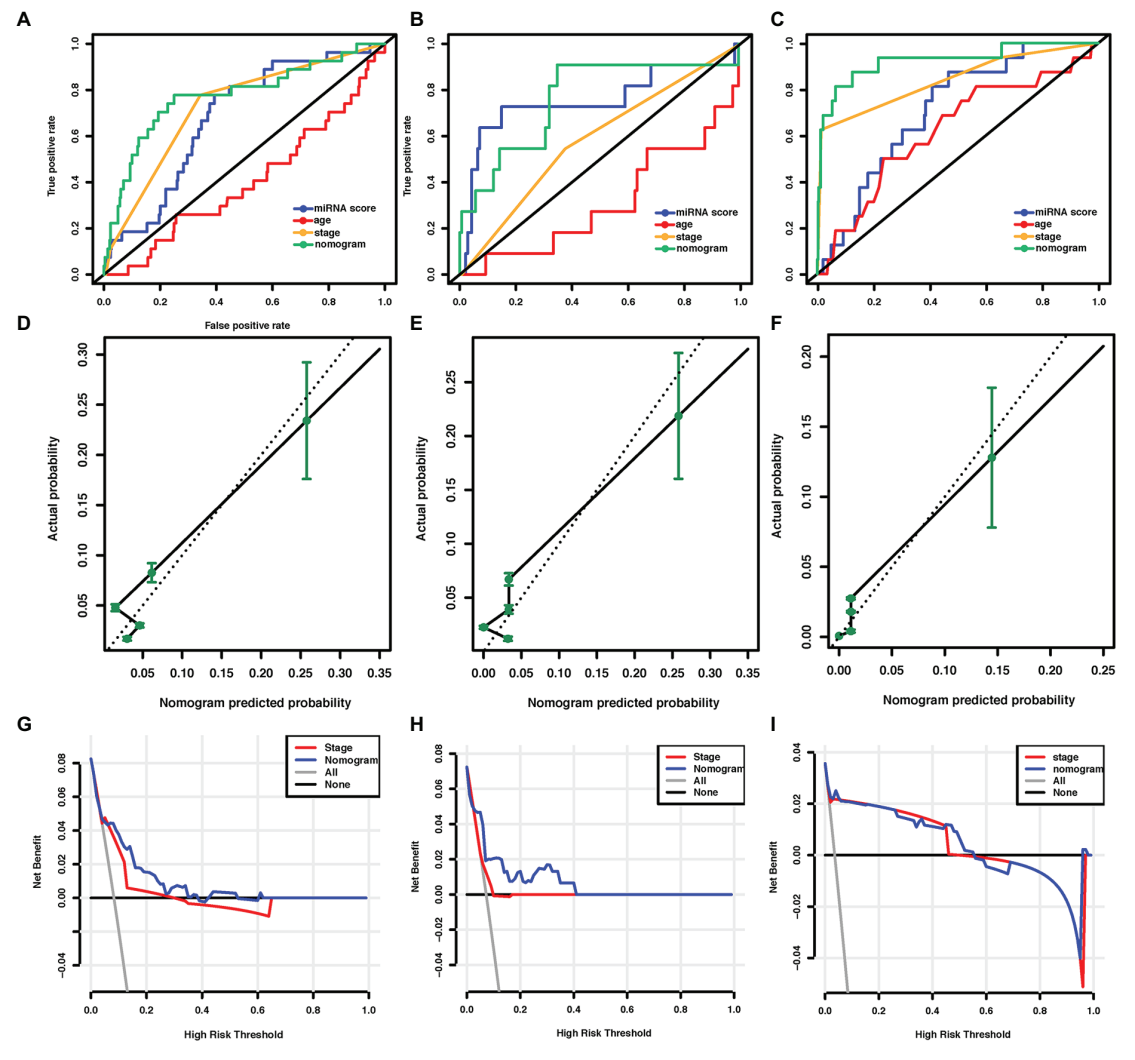
Assessment of the miRNA-based nomogram. Receiver operating characteristic (ROC) curves of eight-miRNA signature, age at diagnosis, stage, and the miRNA-based nomogram model predicting LM in **(A)** training cohort, **(B)** internal validation cohort, and **(C)** external validation cohort. Calibration plots for miRNA-based nomogram model predicting LM in the **(D)** training cohort, **(E)** internal validation cohort, and **(F)** external validation cohort. Calibration curves depict the calibration of the model in terms of the agreement between the predicted risks of LM and the observed outcomes of LM. The y-axis represents the actual LM rate. The x-axis represents the predicted LM risk. The dashed line (the 45-degree diagonal line) represents a perfect prediction by an ideal model, and the black solid line represents the performance of the nomogram of which a closer fit to the diagonal dotted line represents a better prediction. Decision curve analysis of the miRNA-based nomogram model and tumor staging system in **(G)** training cohort, **(H)** internal validation cohort, and **(I)** external validation cohort. The y-axis displays the net benefit. Solid black line: net benefit when all breast cancer patients are considered as not having the LM; solid gray line: net benefit when all breast cancer patients are considered as having LM. Solid red line: net benefit when all breast cancer patients are considered according to the tumor staging system. Solid blue line: net benefit when all breast cancer patients are considered according to the miRNA-based nomogram model. The net benefit was calculated by subtracting the proportion of all patients who are false positive from the proportion who are truly positive, weighting by the relative harm of giving up treatment compared with the negative consequences of unnecessary treatment (Vickers et al., 2008). miRNA, microRNA; ROC, receiver operating characteristic; LM, lung metastasis; and BC, breast cancer.

In the training cohort, according to the results of univariate logistic regression analysis, the eight-miRNA signature, and five clinical risk factors (age at diagnosis, tumor size, grade, TNM stage, and HER2 status) with values of *p* less than 0.1 were included in multivariate regression analysis for assessing the independent risk factors for lung metastasis (Table 2). A multivariate logistic regression analysis was used to develop a nomogram model and found age at diagnosis, TNM stage, and the eight-miRNA signature significantly increased the likelihood of lung metastasis (Figure 3). The AUC of the miRNA-based nomogram model was 0.774 (95% CI, 0.669–0.879) in the training cohort (Table 3; Figure 4A). The calibration curve of the miRNA-based nomogram was very close to the standard 45-degree diagonal line, which showed good calibration in the training cohort (Figure 4D).

**Table 2:** Risk factors for lung metastasis (LM) in training cohort.

	Univariate analysis		Multivariate analysis	
	OR (95% CI)	*p* value	OR (95% CI)	*p* value
miRNA score	1.898 (1.237–2.912)	0.0033	1.651 (1.046–2.606)	0.0311
Age at diagnosis	0.583 (0.330–1.020)	0.0587	0.486 (0.275–0.862)	0.0134
Tumor size	1.499 (1.148–1.958)	0.0030		
Grade	3.129 (0.824–11.884)	0.094		
TNM stage	3.494 (1.905–6.407)	<0.0001	4.025 (2.078–7.795)	<0.0001
ER status	0.738 (0.298–1.824)	0.511		
PR status	1.085 (0.487–2.416)	0.842		
HER2 status	2.759 (1.087–7.005)	0.0328		
Hormone therapy	0.563 (0.253–1.254)	0.1599		

LM, lung metastasis; miRNA, microRNA; ER, estrogen receptor; PR, progesterone receptor; HER2, human epidermal growth factor receptor 2; and TNM, the tumor node metastasis.

**Table 3:** Area under the receiver operating characteristics curve (AUC) of prognostic indicators for lung metastasis in breast cancer (BC).

Variables	Training cohort	Internal validation cohort	External validation cohort
miRNA score	0.681 (95% CI, 0.589–0.774)	0.754 (95% CI, 0.561–0.946)	0.711 (95% CI, 0.608–0.815)
Age at diagnosis	0.403 (95% CI, 0.290–0.516)	0.282 (95% CI, 0.117–0.448)	0.623 (95% CI, 0.479–0.768)
TNM stage	0.727 (95% CI, 0.628–0.825)	0.583 (95% CI, 0.407–0.759)	0.840 (95% CI, 0.716–0.963)
Nomogram model	0.774 (95% CI, 0.669–0.879)	0.763 (95% CI, 0.597–0.929)	0.925 (95% CI, 0.846–1.000)

TNM, the tumor node metastasis; AUC, area under the receiver operating characteristics curve.

### Assessment of the Eight-miRNA Signature and Nomogram Model in Validation Cohorts

We then examined the predictive ability of our eight-miRNA signature and nomogram model in two validation cohorts. The distributions of the miRNA-based risk score, OS, OS status, and the expression profiles of predictive miRNAs in the internal validation cohorts have been shown in Supplementary Figure S3A. The eight-miRNA signature and miRNA-based nomogram model displayed an AUC of 0.754 (95% CI, 0.561–0.946) and 0.763 (95% CI, 0.597–0.929) for lung metastasis risk prediction, respectively (Table 3; Figure 4B). The calibration curve of the miRNA-based nomogram also exhibited favorable accordance between the predicted risk and the actual risk in the internal validation cohort (Figure 4E).

**Figure 5:**
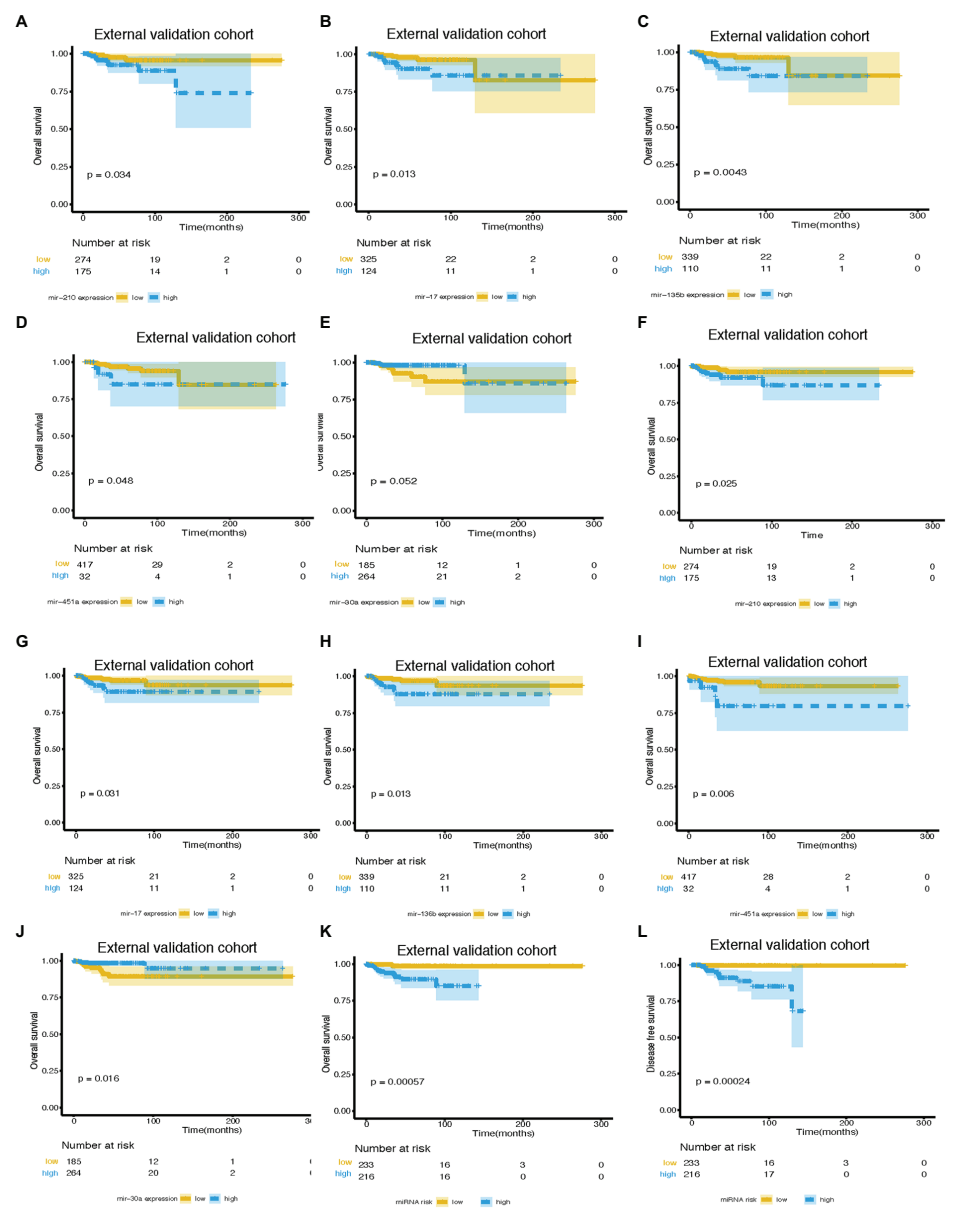
Survival curves of BC patients stratified by different variables. KM curves of overall survival of breast cancer patients stratified by **(A)** mir-210 expression, **(B)** mir-17 expression, **(C)** mir-135b expression, **(D)** mir-451a expression, **(E)** mir-30a expression, and **(K)** miRNA risk score in the external validation cohort. Kaplan-Meier curves of disease-free survival of breast cancer patients stratified by **(F)** mir-210 expression, **(G)** mir-17 expression, **(H)** mir-135b expression, **(I)** mir-451a expression, **(J)** mir-30a expression, and **(L)** miRNA risk score in the external validation cohort. miRNA, microRNA; BC, breast cancer.

An independent external validation cohort of 449 patients who fulfilled the same requirements as above was recruited from the TCGA dataset. A total of seven of the eight miRNAs identified in our study were found in the TCGA miRNA dataset (the exception being miR-663). The distributions of the miRNA-based risk score, OS, OS status, and the expression profiles of predictive miRNAs in the external validation cohorts has been shown in Supplementary Figure S3B. Among them, the elevated expression of four miRNAs was significantly associated with poorer OS and disease-free survival (DFS) (miR-210, miR-451a, miR-135b, and miR-17) (Figures 5A–D,F–I). In the meantime, the higher expression of miR-30a indicated better OS and DFS (Figures 5E,J). Due to the different sequence platforms used in the external validation cohort, the risk score of the external validation cohort was constructed using seven miRNAs. An optimal cut-off point was determined by ROC to dichotomize the samples into low and high-risk groups. Patients with higher miRNA risk scores tended to have a poorer prognosis than those with lower risk scores (Figures 5K,L). Other than predicting OS and DFS, the miRNA risk score was also significantly associated with the risk of lung metastasis in univariate and multivariate logistic regression analysis (Table 4). The miRNA signature and miRNA-based nomogram model displayed an AUC of 0.711 (95% CI, 0.608–0.815) and 0.925 (95% CI, 0.846–1.000) for the estimation of lung metastasis risk respectively (Table 3; Figure 4C). The calibration plot showed that the predicted risks of the nomogram were in good accordance with the actual risks (Figure 4F).

**Table 4:** Risk factors for lung metastasis in external validation cohort.

	Univariate analysis		Multivariate analysis	
	OR (95% CI)	*p* value	OR (95% CI)	*p* value
miRNA score	2.748 (1.299–5.816)	0.0082	4.207 (1.440–12.290)	0.0086
Age at diagnosis	1.678 (0.861–3.268)	0.1277	1.748 (0.811–3.769)	0.1540
TNM stage	29.345 (9.153–94.086)	<0.0001	32.540 (8.986–117.830)	<0.0001

TNM, the tumor node metastasis.

### Comparison With Other Prognostic Markers

Currently, the conventional TNM staging system is the standard tool for risk evaluation in clinical practice. When comparing the AUC, we found that the miRNA-based prediction nomogram achieved better predictive accuracy than the TNM stage in the training cohort and two validation cohorts (Table 3). NRI and IDI were employed to compare the discriminative ability between our model and the TNM stage. Compared the TNM stage alone, the NRI values for miRNA-based prediction nomogram were 0.216 (95% CI, 0.048–0.384, value of *p* = 0.012), 0.307 (95% CI, 0.020–0.594, value of *p* = 0.036) and 0.308 (95% CI, 0.081–0.535, value of *p* = 0.008) in the training cohort and two validation cohorts, respectively (Table 5). The IDI values for miRNA-based prediction nomogram were 0.065 (95% CI, 0.015–0.115, value of *p* = 0.011), 0.093 (95% CI, 0.021–0.165, value of *p* = 0.011), and 0.025 (95% CI, −0.048–0.098, value of *p* = 0.500) in the training cohort and two validation cohorts, respectively (Table 5). Both NRI and IDI indicated a superior predictive ability of our model compared to the TNM staging system.

**Table 5:** The improvement of miRNA-based nomogram in predicting lung metastasis according to net reclassification improvement (NRI) and integrated discrimination improvement (IDI).

Training cohort				Internal validation cohort				External validation cohort			
NRI (95% CI)	*p*	IDI (95% CI)	*p*	NRI (95% CI)	*p*	IDI (95% CI)	*p*	NRI (95% CI)	*p*	IDI (95% CI)	*p*
0.216 (0.048–0.384)	0.012	0.065 (0.015–0.115)	0.011	0.307 (0.020–0.594)	0.036	0.093 (0.021–0.165)	0.011	0.308 (0.081–0.535)	0.008	0.025 (−0.048–0.098)	0.5

NRI, net reclassification improvement; IDI, the integrated discrimination improvement; and P, *p* value.

Decision-curve analysis was conducted to compare the clinical use of our nomogram to that of the TNM staging system (Stewart et al., 2005; Figures 4G–I). The decision curves in both the training and external validation cohorts showed that if the threshold probability was between 0 and 0.60 (in the internal validation cohort, the threshold probability was between 0 and 0.40), using the miRNA-based nomogram to predict lung metastasis added more benefit than treating either all or no patients. DCA also indicated that the net benefit of the miRNA-based nomogram model was comparable, with several overlaps, or even superior to the TNM staging system. Overall, these results suggested the superiority of the miRNA-based nomogram for its lung metastasis predictive performance when compared to the TNM stage.

### Identification of Potential Targets for Predictive miRNAs and Their Roles in Lung Metastasis

**Figure 6:**
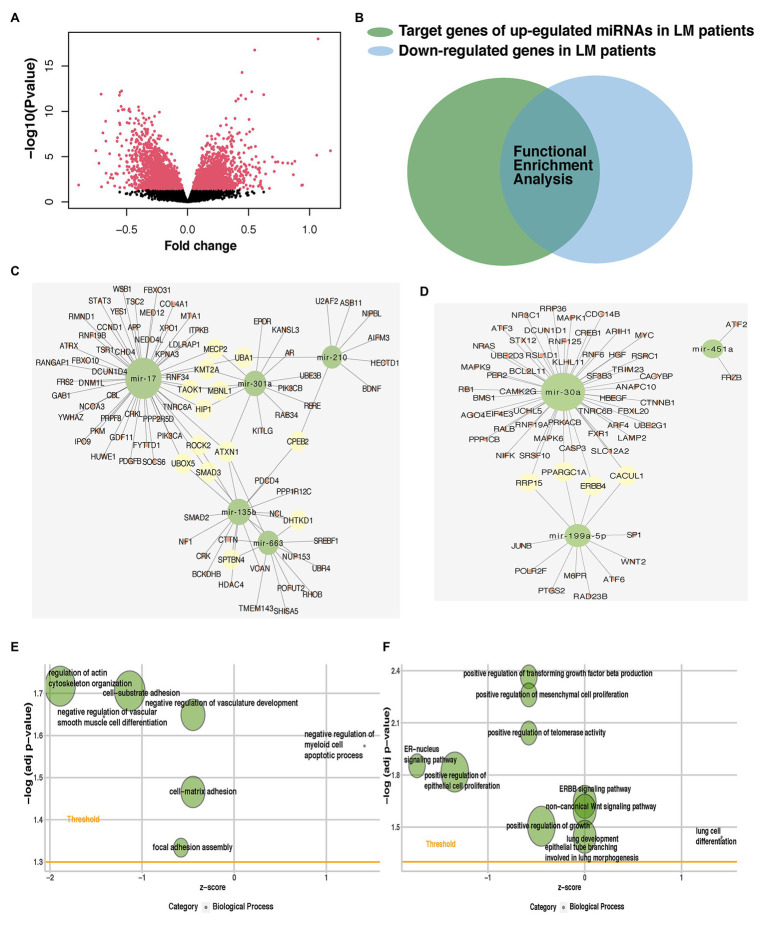
Identification of potential targets for predictive miRNAs and their role in lung metastasis. **(A)** Volcano plot of mRNAs expression in the METABRIC dataset. **(B)** Venn diagram was plotted to show the overlap between differentially expressed mRNAs (DEmRNAs) and gene targets for predictive miRNAs. The overlap of each predictive miRNA was used in subsequent analysis. miRNA-mRNA interaction networks of the hub genes of **(C)** five upregulated or **(D)** three downregulated predictive miRNAs. Enriched metastasis-related gene ontology (GO) terms of the hub genes of **(E)** five upregulated or **(F)** three downregulated predictive miRNAs. miRNA, microRNA; METABRIC, molecular taxonomy of breast cancer international consortium; DEmRNAs, differentially expressed mRNAs; and GO, gene ontology.

We identified the gene targets for predictive miRNAs using *in silico* predictions (TargetScan, miRWalk, and miRDB) and experimentally verified microRNA database (miRTarBase). We also acquired matched mRNA transcriptome data of the patients enrolled in the analysis of identifying DEmiRNAs. Around 3,791 genes were differentially expressed, of which 1,710 were upregulated and 2,081 were downregulated (Figure 6A; Supplementary Table S3). The benefit of using matched mRNA dataset was that it acted as an approach to be the functional validation of targets genes identified by the prediction algorithm (Krishnan et al., 2015). We further used Venn diagram to found the overlap between DEmRNAs and the gene targets for miRNAs and proceeded to the subsequent analysis (Figure 6B; Supplementary Table S4).

Gene ontology and Kyoto Encyclopedia of Genes and Genomes pathway enrichment analyses were performed for the overlapped target genes of each predictive miRNAs. Among pro-metastatic miRNAs, miR-17 mainly interfered with cell cycle arrest (BP), mitotic G1/S transition checkpoint (BP), positive regulation of autophagy (BP), signal transduction by p53 class mediator (BP), focal adhesion (KEGG), signaling pathways regulating pluripotency of stem cells (KEGG), regulation of actin cytoskeleton (KEGG), and hippo signaling pathway (KEGG; Supplementary Figures S4A,B). miR-210 negatively influenced lactate metabolic process (BP), post-embryonic animal organ development (BP), and negative regulation of vascular permeability (BP; Supplementary Figure S4C). Another pro-metastatic miR-663 potentiated the invasion of tumor cells by targeting actin filament polymerization (BP), cell-substrate junction assembly (BP), cell-substrate junction assembly (BP), focal adhesion assembly (BP), and actin filament organization (BP; Supplementary Figure S4D). The protective miR-30a was found able to restrain PI3K-Akt signaling pathway (KEGG), Ras signaling pathway (KEGG), IL-17 signaling pathway (KEGG), estrogen signaling pathway (KEGG), MAPK signaling pathway (KEGG), Wnt signaling pathway (KEGG), and ERBB signaling pathway (KEGG; Supplementary Figure S4E). No terms were enriched in the enrichment analysis of other miRNAs alone.

These miRNAs functioned together in the organism, so then we tried to identify the role of five upregulated or three downregulated miRNAs as a whole. Hub genes of the target genes for five upregulated or three downregulated miRNAs were generated to identify central elements of pro-metastatic and anti-metastatic biological networks (Supplementary Table S5). miRNA-mRNA interaction networks of the hub genes of five upregulated or three downregulated miRNAs were plotted (Figures 6C,D). The metastatic cascade is composed of a series of sequential events that involve cell detachment from the primary tumor, invasion of these cells into surrounding tissue, intravasation migration, arrest, and extravasation into distant tissues, and formation of metastasis (Lambert et al., 2017). GO analysis was also performed for the hub genes of five upregulated or three downregulated miRNAs (Figures 6E,F; Supplementary Table S6). We found our predictive miRNAs participated in most of the above events and thereby promoting lung metastasis. They suppressed the adhesion between cancer cells and matrix facilitated the vasculature development and hematogenous metastasis, promoted proliferation, and then adapted to the lung so as to form the metastasis.

### miR-30a and miR-135b Have Unique Roles in Lung Metastasis of BC

**Figure 7:**
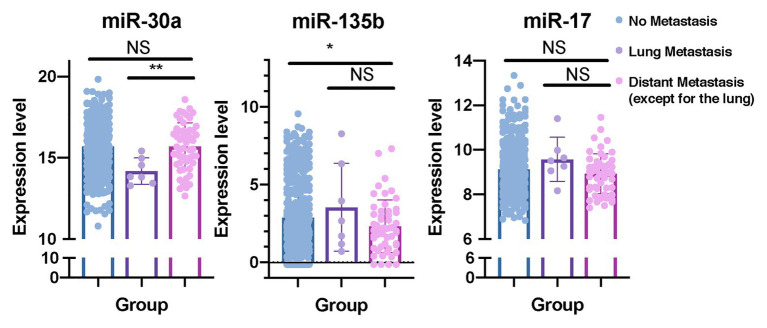
miR-30a and miR-135b have unique roles in lung metastasis of BC. Dot plots were plotted to show the distributions of miR-30a, miR-135b, and miR-17 in BC patients with distant metastasis except for the lung, BC patients with lung metastasis only, and BC patients without metastasis. miRNA, microRNA; BC, breast cancer. ^*^p < 0.05; ^**^p < 0.01.

In order to determine whether these eight predictive miRNAs were unique to lung metastasis in BC patients, we first identified DEmiRNAs between patients with lung metastasis only and patients with distant metastasis except for the lung (Supplementary Tables S7, S8). Baseline clinical and pathological characteristics of the study participants in the comparison were listed in Table 6. Compared to patients with distant metastasis except for the lung, protective miR-30a was found to be downregulated in patients with lung metastasis only. On the contrary, miR-135b was upregulated in patients with lung metastasis only. In addition, we recognized DEmiRNAs between patients with distant metastasis except for the lung and patients without metastasis (Table 6; Supplementary Tables S7, S8). The expression levels of miR-135b and miR-17 were downregulated in patients with distant metastasis except for the lung. In order to further confirm whether these three miRNAs were lung-metastasis-specific in BC patients, we performed dot plots to see their expression levels in patients with distant metastasis except for the lung, patients with lung metastasis only, and patients without metastasis (Figure 7). The expression level of miR-30a was extremely low in BC patients with lung metastasis, while the expression level of miR-135b was extremely high in BC patients with lung metastasis. These analyses of identifying DEmiRNAs in different subgroups of BC patients showed the unique roles of miR-30a and miR-135b in lung metastasis of BC.

**Table 6:** Demographics of the samples recruited in subgroup analysis.

Variables	lung metastasis only (*n* = 6)	distant metastasis except for the lung (*n* = 54)	without metastasis (*n* = 433)
Median age at diagnosis in years (IQR)	65 (56–71.5)	57 (47–63.25)	60 (50.5–67.00)
Median follow up time from diagnosis in days (IQR)	1,233 (645.3–3,578)	1,096 (190.5–2,405)	343.5 (109.3–1,064)
Pam50 subtype			
Luminal A	0	22	201
Luminal B	1	11	64
HER2	0	5	21
Basal like	2	6	94
Normal breast-like	1	2	13
Unknown	2	8	40
TNM stage			
1	0	7	151
2	2	27	276
3	4	14	5
4	0	6	1
ER status			
Positive	1	37	115
Negative	5	12	297
Unknown	0	5	21
PR status			
Positive	1	31	266
Negative	5	19	144
Unknown	0	4	23
HER2 status			
Positive	1	2	52
Negative	1	17	241
Unknown	4	35	140
Menopausal state			
Pre	1	12	82
Post	5	32	299
Peri	0	2	19
Unknown	0	8	33
Patient metastatic sites			
Lung	*6*	*0*	*0*
Bone	*0*	*29*	*0*
Brain	*0*	*3*	*0*
Liver	*0*	*7*	*0*
Multi-organ Metastasis	*0*	*15*	*0*
No metastasis	*0*	*0*	*433*
Vital status			
Alive	1	16	433
Dead	5	38	0

PAM50, prediction analysis of microarray 50; ER, estrogen receptor; PR, progesterone receptor; HER2, human epithelial growth factor receptor 2; and TNM, the tumor node metastasis.

### Pan-Cancer Analysis of the Expression Levels of Eight Predictive miRNAs in Patients With Lung Metastasis and Without Metastasis

We performed differential miRNA expression analyses between patients with lung metastasis and patients without metastasis in six cancer types (ACC, BLCA, SARC, SKCM, CESC, and STAD; Supplementary Tables S9, S10). The expression level of miR-663 was not detected in these datasets. The distributions of the expression levels of these predictive miRNAs in six cancer types were also presented (Supplementary Figures S5A–F). Combined analyses indicated that compared to patients without metastasis, miR-210 was upregulated in ACC and SARC patients with lung metastasis. The expression level of miR-199a-5p was higher in BLCA patients with lung metastasis, whereas the expression level of miR-199a-5p was lower in SARC patients with lung metastasis. miR-17 was upregulated in SARC patients with lung metastasis. Elevated expression levels of miR-135b were detected in ACC patients with lung metastasis. Compared to patients without metastasis, the expression level of miR-30a was suppressed in ACC patients with lung metastasis.

## Discussion

Based on Surveillance, Epidemiology, and End Results (SEER) database, the median survival time for BC patients with lung metastases was 21 months, and only 15.5% of the patients were alive for more than 3 years (Xiao et al., 2018). Once metastasis occurs, the disease is largely incurable. Identifying effective predictive biomarkers to construct an accurate nomogram model to predict the lung metastasis status of BC patients is an advisable choice applied in the clinical practice. At present, the TNM staging system is commonly used to assess the metastasis probability of BC patients. But as discussed above, a single clinical parameter has limited power of outcome prediction. We put forward the idea for the first time that BC patients with lung metastasis might have unique clinicopathologic characteristics and miRNA expression profiles, which could distinguish themselves from those who had no lung metastasis.

Subgroup analysis suggested that miR-30a and miR-135b have distinct roles in lung metastasis of BC patients. miR-30 has been reported to be able to stabilize pulmonary vessels and inhibit pulmonary vascular hyperpermeability in the premetastatic phase (Qi et al., 2015). The role of miR-135b in BC patients remains controversial. miR-135b reduces the proliferation of ERα-positive BC cells (Aakula et al., 2015), but promotes the proliferation and invasion of triple-negative breast cancer (TNBC) by downregulating *APC* expression (Lv et al., 2019). TNBC especially tends to metastasize to the lungs (Foulkes et al., 2010), which may partly explain the uniqueness of miR-135b to the lung metastasis. The precise roles of these miRNAs in the lung have been studied to some extent, yet further research is needed to fill the gap.

The significance of miRNAs is better appreciated from the aspect of their potential functional impact on biological pathways, as these influence the outcomes for the patient (Krishnan et al., 2015). Cancer metastasis is a complicated process, and the outcome of metastasis depends on the interactions between cancer cells and a given microenvironment. We could see that the targets for the identified miRNAs were enriched for cell proliferation, invasion, and migration, which participated in the whole regulatory process of metastasis. During lung metastasis, metastatic tumor cells will rewrite their biology and expression profiles to adapt to the distant microenvironment, which endows tumor cells with full competence for outgrowth in the lung. Therefore, we also identified some adaptations specific to the lung microenvironment. The target of miR-30a, *SEMA3A*, has been reported to modulate distal pulmonary epithelial cell development and alveolar septation, which has also been found upregulated in patients with lung metastasis (Becker et al., 2011). Transforming growth factor beta (TGFβ) promotes metastasis of BC to the lungs but it is dispensable to bone metastasis (Chen et al., 2018). We identified “positive regulation of TGFβ production” enriched in patients with lung metastasis. Terms concerning lung such as “lung development” and “epithelial tube branching involved in lung morphogenesis” have also been identified in GO analysis.

We also conducted a pan-cancer analysis to figure out whether the eight predictive miRNAs were specific to BC. Some of the miRNAs had consistent effects in different cancer types, such as miR-30a, miR-17, miR-451a, and miR-135b, while others showed controversial effects, such as miR-210, miR-301a, and miR-199a. Previous studies also identified the role of these predictive miRNAs in lung metastasis of other types of cancer (Qi et al., 2015; Kai et al., 2016; Jin et al., 2017; Xu et al., 2019; Wang et al., 2020). miR-17, miR-135b, and miR-210 facilitate cancer cells to metastasize to the lungs, while miR-30a and miR-451a suppress lung metastasis, which exerts similar effects to our results. The lack of research and missing data of miR-663 suggests it can serve as an appealing target for future research. In addition, the notion that miRNAs exert both oncogenic and tumor-suppressive effects has been put forward (Rohan et al., 2019). An individual miRNA could regulate the expression of hundreds of genes. The effect of miRNA in each situation depends on the balance of the pro-tumor and anti-tumor pathways. Multiple biological factors can interfere with the balance, such as the interplay between cells and microenvironment, energy supply, and so on. Although two miRNAs have conflicting roles in pan-cancer analysis, the overall consistency indicated the significant importance of these eight miRNAs in lung metastasis.

The univariate and multivariate logistic regression analysis showed that the eight-miRNA signature could be an independent risk factor in training and validation cohorts. The AUC of eight-miRNA signature alone for lung metastasis prediction showed a little bit smaller than that of the TNM staging system in training and external validation cohort. Therefore, the comprehensive predictive nomogram was constructed integrating the risk score and conventional clinical parameters including stage and age, all of which were verified as an independent risk factor using univariate and multivariate logistic regression analysis for the lung metastasis status of BC patients. Apart from AUC, the calibration plot was also used to assess the discrimination performance of the nomogram model. Although the overall trend was in line with the 45-degree ideal diagonal line, yet the calibration plot showed some deviation, which may due to the limited events and thus affecting the power. NRI, IDI, and DCA were used to evaluate the prediction ability between miRNA-based nomogram and the TNM staging system. The results of NRI indicated the significant improvement of miRNA-based nomogram in all three cohorts, and the results of IDI suggested that the nomogram model improved the predictive power, yet failed to reach a significant level in the external validation cohort. DCA results also indicated that our miRNA-based nomogram improved current treatment standards, while the ideal model was the model with the positive net benefit at any given threshold.

However, several limitations of our study should be acknowledged. Firstly, due to the different sequence platforms, only seven of eight predictive miRNAs were identified in the external validation cohort, so we did not adopt the risk scores and cut-off points generated in the training set as previous research suggested (Volinia and Croce, 2013; Krishnan et al., 2015; Rohan et al., 2019). Secondly, the limited number of events in the cohorts may affect the statistical power. Among DEmiRNAs that were not selected by LASSO method, some have also been reported to be related to lung metastasis (Ma et al., 2010). HER2 overexpression has been proved to be a risk for the development of visceral-only metastasis including lung (Bartmann et al., 2017). However, HER2 status reached a significant level in univariate logistic regression but failed in multivariate logistic regression, so it was not included in the nomogram model. Last but not least, we have emphasized the complexity of miRNA regulation previously. Therefore, experiments for revealing and verification of their roles in lung metastasis are crucial in the future.

In this study, we constructed a nomogram model based on multiple lung metastasis-related miRNAs and clinical risk factors to predict the lung metastasis of BC patients. We screened the high-throughput sequence data from the METABRIC database to explore DEmiRNAs and used the LASSO method to identify an eight-miRNA signature. The risk score was calculated by the multivariate logistic coefficient multiplied by the expression of the miRNA. Then the risk score and clinical risk factors were combined together to construct a miRNA-based nomogram, which was assessed by the calibration plot, ROC analysis, NRI, IDI, and DCA. Internal and external validation was also performed to evaluate the nomogram model. Functional enrichment analyses were performed to identify the potential biological roles of eight predictive miRNAs. Subgroup analysis of BC patients with different distant metastasis showed that miR-30a, miR-135b, and miR-17 have unique roles in lung metastasis of BC. Pan-cancer analysis of patients with lung metastasis or without metastasis in six types of cancer indicated the significant importance of eight predictive miRNAs in lung metastasis. A biomarker-based approach to accurately predict the metastasis status of BC patients is urgently needed in the era of precision medicine. Risk assessment is vital for making appropriate therapeutic decisions and follow-up strategies in BC patients. If a patient has a high probability to have lung metastasis in the future, we might recommend the patient to take a close inspection of the lung and adopt advanced treatment. This model might be able to perform well in all patients, for it was constructed based on large-scale datasets. In addition, this risk score was also a significant factor in affecting survival. Therefore, this nomogram could be used as a supportive graphic tool in clinical practice to facilitate treatment decisions of BC patients.

## Conclusion

In our current study, we identified eight predictive miRNAs from publicly available data and constructed an eight-miRNA based nomogram that incorporated other clinical parameters including stage and age to predict the lung metastasis status of BC patients, whose prediction power was better than that of conventional TNM stage system. Subgroup analysis suggested that miR-30a, miR-135b, and miR-17 may have unique roles in lung metastasis of BC patients. On the basis of the GO, KEGG enrichment, and pan-cancer analyses, the eight miRNAs played crucial roles in lung metastasis cascade. Therefore, our eight-miRNA-based nomogram might be a vital tool for lung metastasis prediction in BC patients, aiding in developing personalized treatment strategies.

## Data Availability Statement

The datasets analyzed for this study can be found in The Cancer Genome Atlas (https://portal.gdc.cancer.gov/) and European Genome Archive (https://ega-archive.org/).

## Ethics Statement

Ethical review and approval was not required for the study on human participants in accordance with the local legislation and institutional requirements. Written informed consent for participation was not required for this study in accordance with the national legislation and the institutional requirements.

## Author Contributions

LZ initiated and organized the study. LZ and JP designed and carried out bioinformatics and statistical analyses, drew figures, and drafted the manuscript. ZW, CY, and JH participated in editing the manuscript. All authors contributed to the article and approved the submitted version.

### Conflict of Interest

The authors declare that the research was conducted in the absence of any commercial or financial relationships that could be construed as a potential conflict of interest.

## Figures and Tables

**Table:** 

BC	Breast cancer
miRNAs	MicroRNAs
METABRIC	Molecular taxonomy of breast cancer international consortium
DEmiRNAs	Differentially expressed miRNAs
TCGA	The cancer genome atlas
AUC	Area under the receiver operating characteristics curve
TNM	The tumor node metastasis
NRI	Net reclassification improvement
IDI	The integrated discrimination improvement
DCA	Decision-curve analysis
LASSO	Least absolute shrinkage and selection operator
OS	Overall survival
3' UTR	3' untranslated region
KM	Kaplan-Meier
ROC	Receiver operating characteristic curve
ER	Estrogen receptor
PR	Progesterone receptor
HER2	Human epidermal growth factor receptor 2
DEmRNAs	Differentially expressed mRNAs
GO	Gene ontology
KEGG	Kyoto encyclopedia of genes and genomes
DFS	Disease-free survival
SEER	Surveillance, epidemiology, and end results
EMT	Epithelial-mesenchymal transition
TGFβ	Transforming growth factor beta
ACC	Adrenocortical carcinoma
BLCA	Bladder urothelial carcinoma
SARC	Sarcoma
SKCM	Skin cutaneous melanoma
CESC	Cervical squamous cell carcinoma and endocervical adenocarcinoma
STAD	Stomach adenocarcinoma	LM	Lung metastasis

## Footnotes

^1^https://www.ebi.ac.uk/ega/home

^2^https://portal.gdc.cancer.gov/

^3^http://mirwalk.umm.uni-heidelberg.de/

^4^http://www.mirdb.org/

^5^http://www.targetscan.org/vert_72/

^6^http://mirtarbase.cuhk.edu.cn/php/index.php

^7^https://www.ebi.ac.uk/ega/home

^8^http://cytoscape.org

^9^http://www-01.ibm.com/software/uk/analytics/spss/

^10^http://www.Rproject.org
