# Supplementary material for: Construction of a MicroRNA-Based Nomogram for Prediction of Lung Metastasis in Breast Cancer Patients
Source: Front Genet. 2021 Feb 19;11:580138. doi: 10.3389/fgene.2020.580138 (PMC7933652; doi:10.3389/fgene.2020.580138)
Supplement: Supplementary file 1 [file Data_Sheet_1.zip › supplementary data/supplementary figures/Supplementary Figure Legends.docx]

**Figure S1**

Pearson correlation analysis of 20 most significant up- and down-regulated miRNAs.

miRNA, microRNA.

**Figure S2**

Kaplan–Meier curves of overall survival of breast cancer patients stratified by age at diagnosis.

**Figure S3**

The distributions of risk score, overall survival, vital status, and the expression profiles of predictive miRNAs in the **(A)** internal validation cohort and **(B)** external validation cohort.

miRNA, microRNA.

**Figure S4**

Enriched metastasis-related GO terms of the target genes for **(A)** miR-17, **(C)** miR-210, and **(D)** miR-663. Enriched metastasis-related KEGG pathway of the target genes for **(B)** miR-17 and **(E)** miR-30a.

miRNA, microRNA; GO: gene ontology; KEGG, Kyoto encyclopedia of genes and genomes.

**Figure S5**

Dot plots were plotted to show the distributions of the predictive miRNAs in patients of **(A)** ACC, **(B)** BLCA, **(C)** SARC, **(D)** STAD, **(E)** CESC, **(F)** SKCM with or without lung metastasis.

miRNA, microRNA; ACC, adrenocortical carcinoma; BLCA, bladder urothelial carcinoma; SARC, sarcoma; SKCM, skin cutaneous melanoma; STAD, stomach adenocarcinoma; CESC, cervical squamous cell carcinoma and endocervical adenocarcinoma.
