# Supplementary figures and images for: Construction of a MicroRNA-Based Nomogram for Prediction of Lung Metastasis in Breast Cancer Patients
Source: Front Genet. 2021 Feb 19;11:580138. doi: 10.3389/fgene.2020.580138 (PMC7933652; doi:10.3389/fgene.2020.580138)

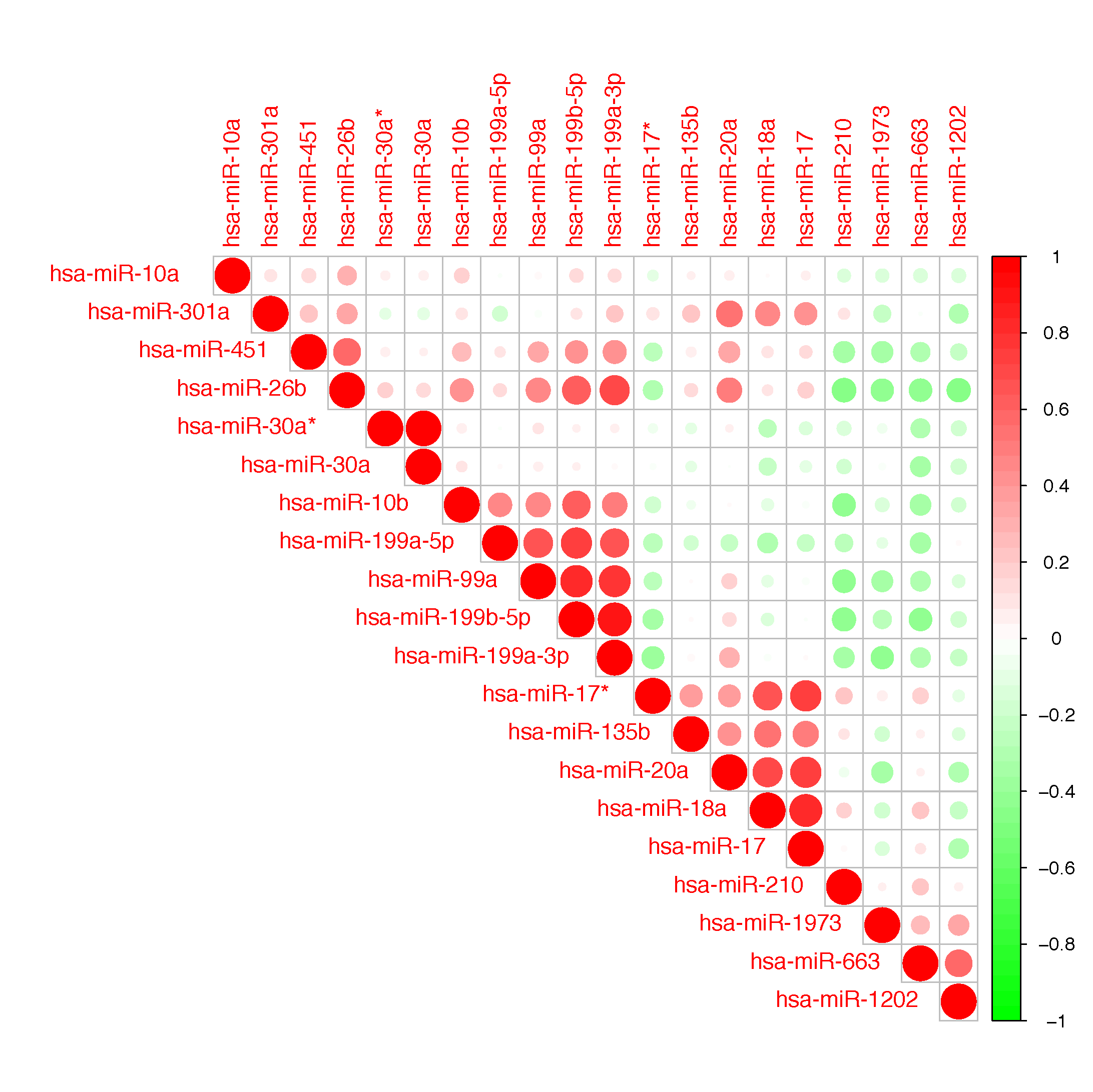

Supplement: Supplementary file 1 [file Data_Sheet_1.zip › supplementary data/supplementary figures/Supplementary Figure 1.tif]

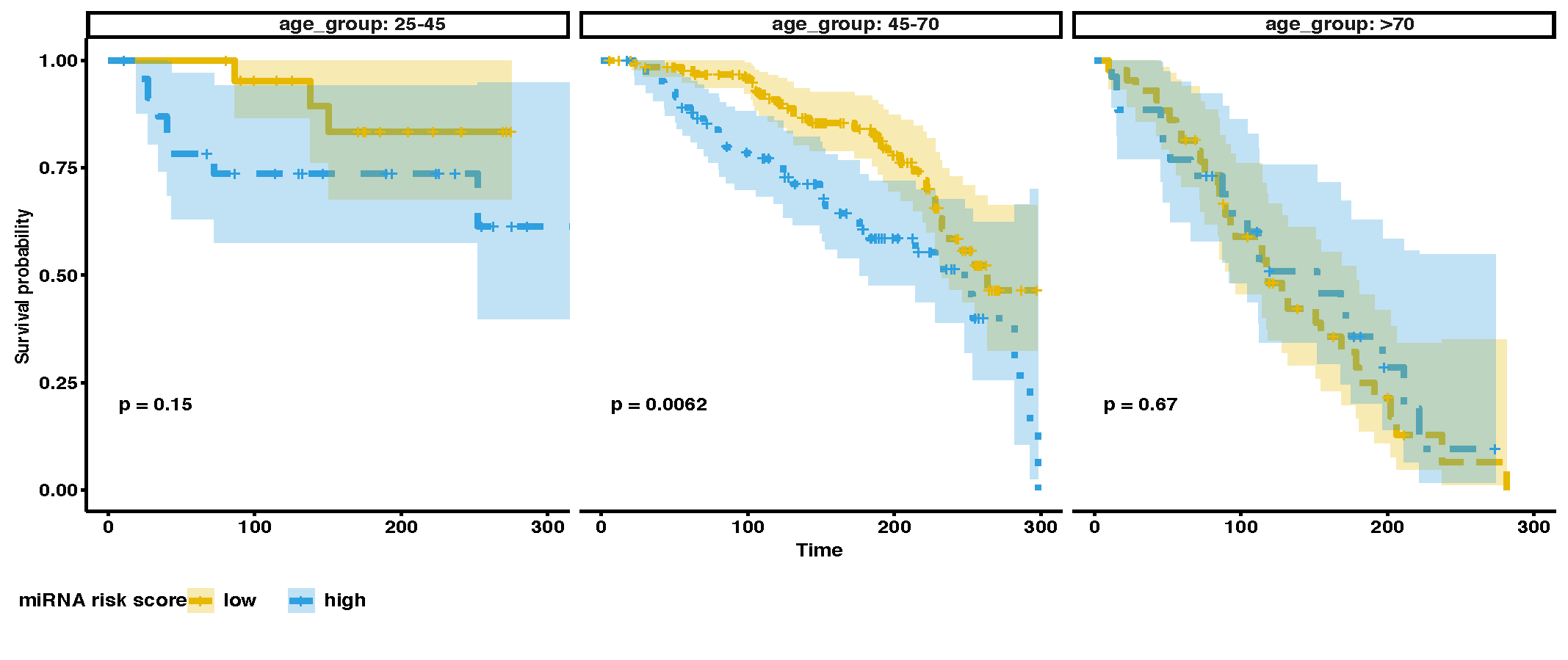

Supplement: Supplementary file 1 [file Data_Sheet_1.zip › supplementary data/supplementary figures/Supplementary Figure 2.tif]

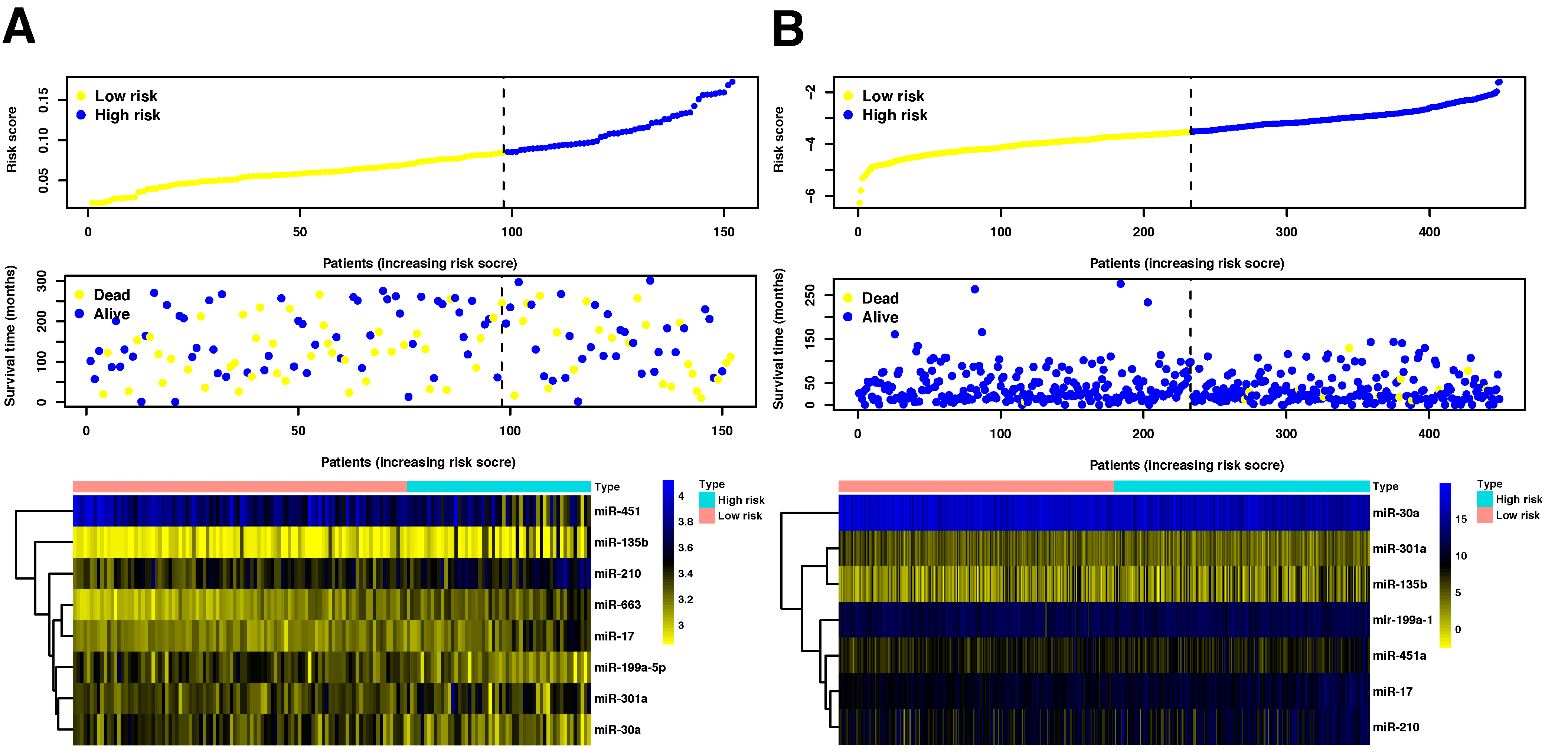

Supplement: Supplementary file 1 [file Data_Sheet_1.zip › supplementary data/supplementary figures/Supplementary Figure 3.tif]

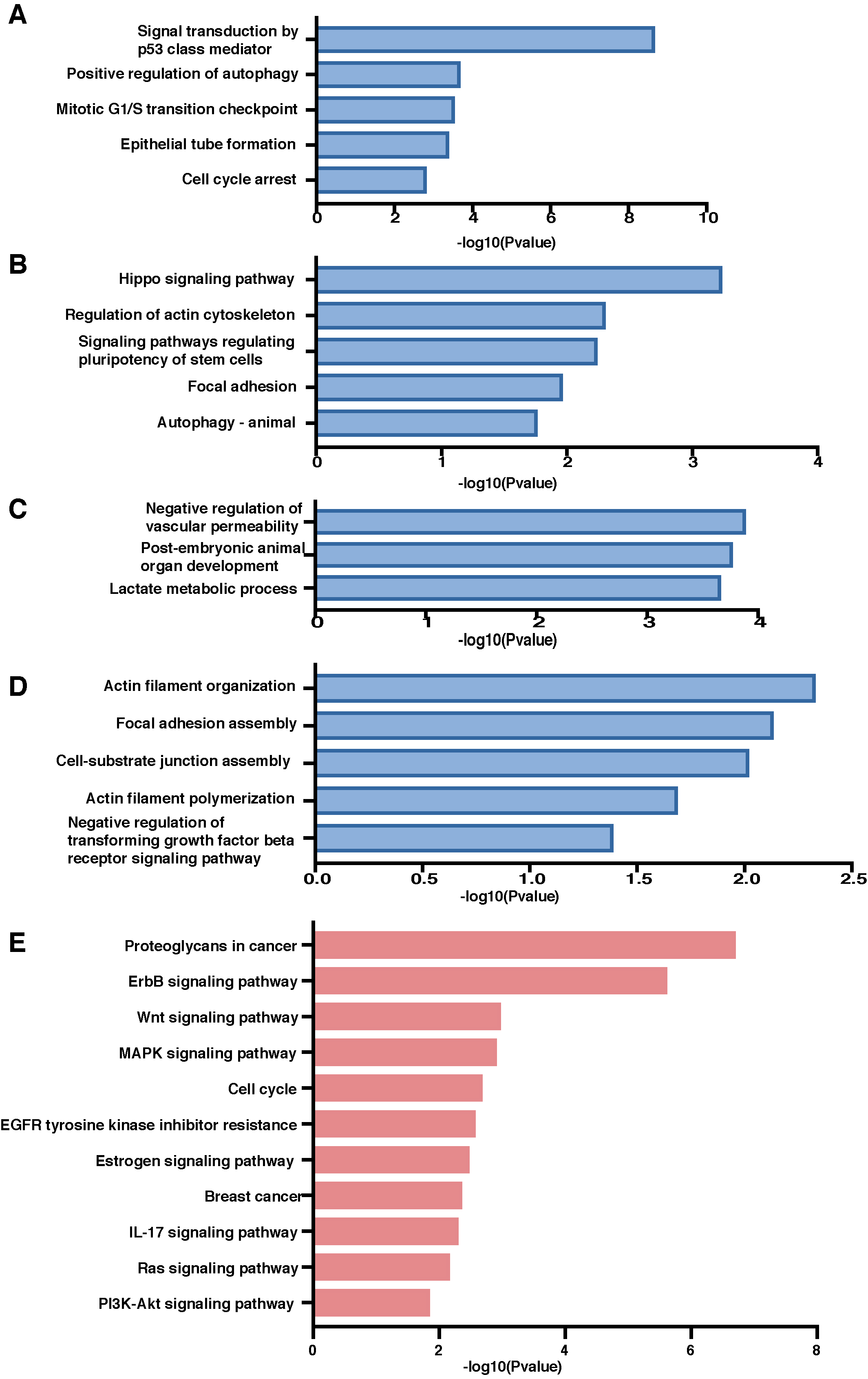

Supplement: Supplementary file 1 [file Data_Sheet_1.zip › supplementary data/supplementary figures/Supplementary Figure 4.tif]

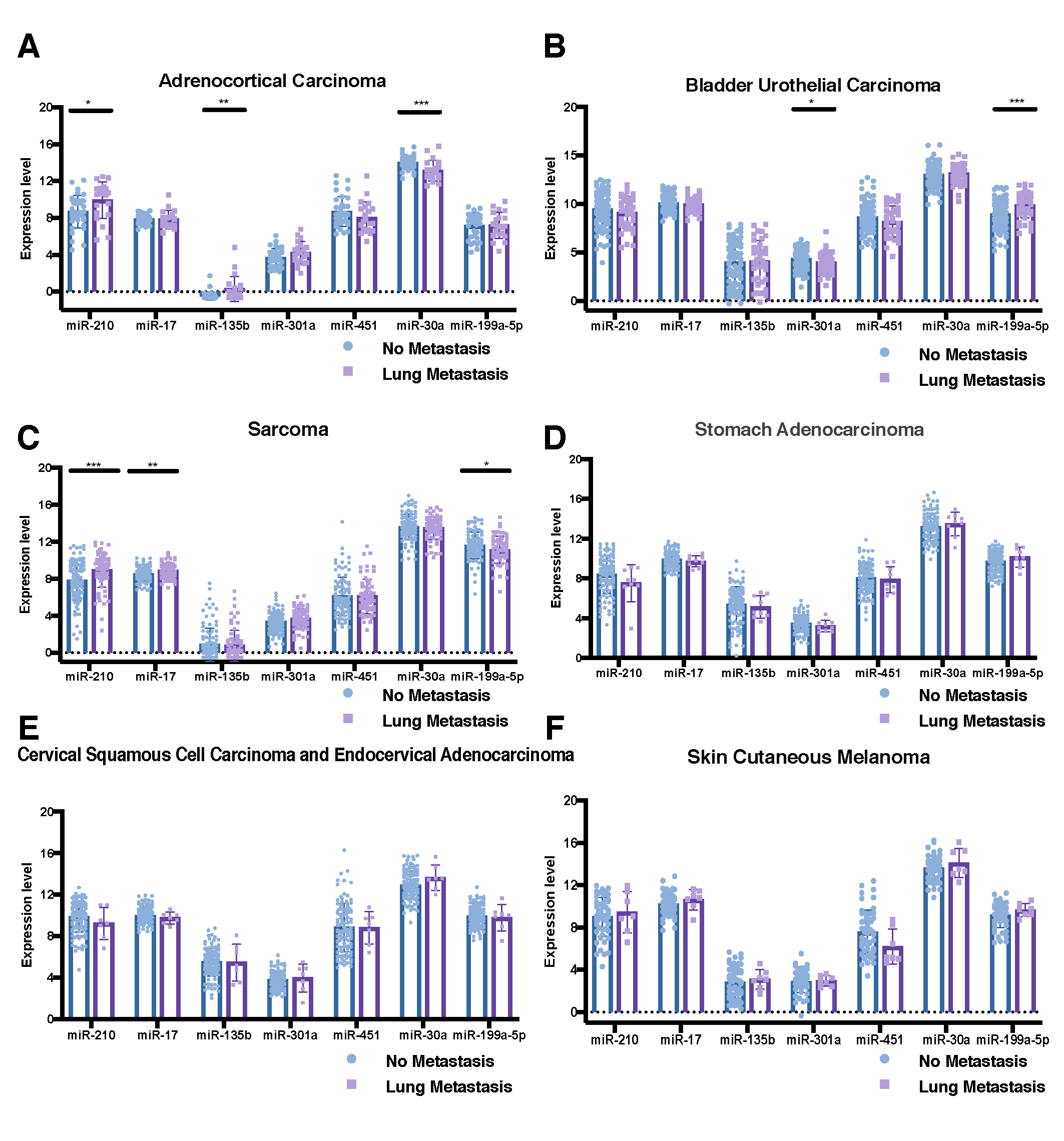

Supplement: Supplementary file 1 [file Data_Sheet_1.zip › supplementary data/supplementary figures/supplementary figure 5.tif]
